# Symptoms and Risk Factors of Ovarian Cancer: A Survey in Primary Care

**DOI:** 10.5402/2012/754197

**Published:** 2012-08-23

**Authors:** Ketan Gajjar, Gemma Ogden, M. I. Mujahid, Khalil Razvi

**Affiliations:** ^1^Department of Obstetrics and Gynaecology, Southend University Hospital NHS Foundation Trust, Westcliff on Sea, Essex SS0 0RY, UK; ^2^Gynaecological Oncology Unit, Royal Preston Hospital, Sharoe Green Lane, Fulwood, Preston, Lancashire PR2 9HT, UK; ^3^Oaklands Surgery, Central Canvey Primary Care Centre, Long Road, Canvey Island, Essex SS8 0JA, UK

## Abstract

In spite of the increased awareness of ovarian cancer symptoms, the predictive value of symptoms remains very low. The aim of this paper is to obtain the views of general practitioners (GPs) in relation to symptom-based detection of ovarian cancer and to assess their knowledge for family history of breast and/or ovarian cancer as a predisposing factor for ovarian cancer. In this questionnaire survey, postal questionnaires were sent to 402 GPs in 132 primary care clinics, out of which we obtained 110 replies (27.4%). Approximately 26% of respondent GPs thought that the symptoms were more likely to be frequent, sudden, and persistent, and one-fifth were unsure of the importance of family history of breast cancer in relation to ovarian cancer. The participant GPs scored a set of symptoms for their relevance to ovarian cancer from 0 (not relevant) to 10 (most relevant). The highest scored symptoms were abdominal swelling (mean ± SD, 8.19 ± 2.33), abdominal bloating (7.01 ± 3.01), and pelvic pain (7.46 ± 2.26). There was a relative lack of awareness for repetitive symptoms as well as gastrointestinal symptoms as an important feature in a symptom-based detection of ovarian cancer.

## 1. Introduction

Ovarian cancer accounts for 4% of all cancers in women, with over 200,000 new diagnoses each year world wide [1]. In early cancers (FIGO Stage 1 and 2), the survival is 80–90% compared with 25% in late cancers (FIGO stage 3 and 4) [2]. However, currently, only 30% of patients are diagnosed in these early stages [1]. No effective screening test exists so the main prospect for early diagnosis is improved identification of symptomatic cancer [3]. The EUROCARE-4 study on cancer survival covering the period up to 2002 reported that England had the worst five-year survival rate for ovarian cancer at 30.2%, with United Kingdom being marginally better, however, still less compared to the European average of 36.5% [4].

Ovarian cancer is accepted as a “silent killer” probably because it is believed that majority of patients are diagnosed in late stage, and that in early stage disease no symptoms are evident. However, over the last decade there has been a lot of research in the symptom-based detection of ovarian cancer [5–9]. It has been shown that the patients with ovarian cancer may have symptoms for at least several months before their diagnosis [5–9]. Additionally, they may experience symptoms more frequently, severely, and persistently than women without the disease [10, 11]. In the UK, patients experiencing unusual symptoms are likely to see their primary care clinician [7], and it is believed that the ovarian cancer symptoms often go unreported and unrecognised for several months before diagnosis [3, 5, 12].

Recognition of certain symptoms by patients and clinicians may identify those suffering from ovarian cancer at an early stage [13, 14]. Awareness of the ovarian cancer symptoms and risks amongst women in general population is low [15]. Additionally, the predictive value of individual symptom in detection of ovarian cancer remains very low and presenting symptoms of ovarian cancer overlaps with those of more common abdominal disease and gastrointestinal disease. The difficulty for the primary care physicians is to decide which patients to be referred urgently for specialist opinion. Not surprisingly, half of the women with ovarian cancer are not referred directly to the gynaecological cancer clinics [11] thus possibly resulting in a delay in the diagnosis. Thus, it is important that the primary care clinicians, who are the first contact for the patients with possible ovarian cancer, are aware of the current research on changing symptomatology of ovarian cancer as well as the risk factors related to ovarian cancer. The objective of the current survey was to assess the knowledge, perception, and understanding of General Practitioners in South East Essex with regard to ovarian cancers.

## 2. Materials and Methods

**Ethical Approval**

As the study reflects opinion of the participants, ethical approval was not required.

**Design**

Questionnaire based survey. The survey was divided into 3 parts. First part included awareness and information access on ovarian cancer. The second part dealt with the symptoms of ovarian cancer. Final part inquired about the management of patient with suspected ovarian cancer and suggestions. We report the results related to the role of symptoms and the family history in detection of ovarian cancer.

**Setting**

General practices (*n* = 132) in South East Essex area.

**Sampling Method**

Purposive sampling, that is, individuals are selected because they met specific criteria (e.g., they are general practitioners in a specific region of UK).

**Sample Size**

We received 110 responses out of 402 GPs from 132 Practices. Questionnaires were sent by post along with a covering letter and a self-addressed stamped return envelope within the study period from July 2008 to June 2009. The covering letter explained the purpose of the survey and gave a brief account of recent advances in ovarian cancer. No reminders were sent. The details of the general practitioners and address of the surgery were obtained from the GP Network. The responses were analysed by the research and audit office. We considered more than 100 responses as adequate to carry out the analysis.

## 3. Results

In this cohort, 66.4% of the respondent GPs were male while 33.6% were female GPs. Majority of the respondents worked full time (77%). About 41% respondents worked in a training practice while 59% respondents worked in a nontraining practice. Out of 108 respondents (2 did not answer the question), 30 had experience of being involved in more than 5 cases of ovarian cancer so far in their career, while 12% of the respondent GPs were never been involved with a case of ovarian cancer. All respondent GPs answered correctly that the smear test does not detect ovarian cancer (Table 1).

Only 6.4% respondent GPs thought that most women diagnosed with early stage disease are reporting symptoms. More than half correctly thought that early clinical diagnosis is possible; however, only one fourth (26.4%) of the respondent GPs thought that women with ovarian cancer are more likely to experience very frequent, sudden onset, and persistent symptoms (Table 1). In a prospective case control study, Goff et al. found that the ovarian cancer patients typically have a symptom frequency of 15 to 30 times per month [7].

When asked about the frequency of symptoms in ovarian cancer (Table 2), only about 20% respondent GPs thought that the symptoms are in frequency of 12–30 times a month. More than two third of the GPs believed that the symptoms are less frequent.

Family history of breast/ovarian cancer is the single greatest risk factor, which explains 5% to 15% of cases of ovarian cancer [16, 17]. When asked about the importance of family history of breast and ovarian cancer, majority (96.4%) of the respondent GPs were aware of the importance of family history of ovarian cancer, while 80.9% were aware that a family history of breast cancer is also important. GPs were asked to score individual symptoms for their relevance to possible ovarian cancer from 0 (not relevant) to 10 (most relevant).

**Figure 1:**
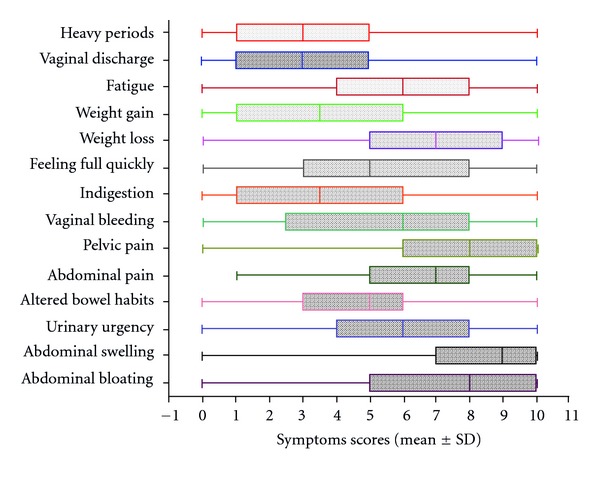
Box-whisker plot showing the mean ± SD of 14 symptoms that are relevant to ovarian cancer on the scale of 0 to 10 (*n* = 92; 0 = not relevant symptom, 10 = most relevant symptom). Abdominal swelling (mean ± SD, 8.19 ± 2.33) is the highest scored symptom while vaginal discharge (3.25 ± 2.9) scored least, suggesting its relatively less relevance to ovarian cancer.

The most important symptoms for GPs when it comes to suspecting ovarian cancer were (see Figure 1) abdominal swelling (mean ± SD, 8.19 ± 2.33), pelvic pain (7.46 ± 2.26), and abdominal bloating (7.01 ± 3.1). These symptoms scored higher than bleeding per vaginum (5.39 ± 3.18), vaginal discharge (3.25 ± 2.9), altered bowel habits (4.5 ± 2.89), and indigestion (3.73 ± 2.98).

## 4. Discussion

Ovarian cancer is not common and one study reported that on an average, a GP is likely to see only one case of ovarian cancer every 5 years in the UK [18]. There has been an increase in the research interest for screening of ovarian cancer with majority of research being focused on use of biomarkers and pelvic ultrasound with multimodal assessments for early detection of ovarian cancer [19]. Over the last decade or so, research has also been intensified on symptom based detection of ovarian cancer with the potential advantage of early detection and timely treatment. A systematic review has estimated that 93% (95% CI: 92% to 94%) of women experienced symptoms before diagnosis of ovarian cancer [20].

The knowledge of symptoms and risk factors of ovarian cancer amongst women in the general population is low [15]; however, it is clear that women with ovarian cancer do experience symptoms and report it to clinicians. A retrospective cohort study of 100 patients from Australia [21] showed that 90% of the patients with early stage disease reported at least one symptom. The challenge for a general practitioner in primary care is to distinguish the symptoms of ovarian cancer from those of other conditions, such as irritable bowel syndrome or other gastrointestinal disease. Few studies have looked at the predictive value of symptoms in detection of ovarian cancer.

In the current survey in primary care, respondent GPs have been able to identify three symptoms as relevant with higher mean symptom scores in comparison to remaining set of 11 other symptoms (Figure 1). These symptoms are abdominal swelling (8.19 ± 2.33), abdominal bloating (7.01  ±  3.01), and pelvic pain (7.46  ±  2.26). However, the mean scores were lower for the gastrointestinal symptoms such as altered bowel habit (4.50 ± 2.89), indigestion (3.73 ± 2.98) and feeling full quickly (5.36 ± 3.07). Thus, GPs in the survey appeared to attach less significance to gastrointestinal symptoms, possibly explaining the reason for higher number of ovarian cancers being referred to other specialties initially. The current observation may suggest that to diagnose the ovarian cancer, equal importance should be given to abdominal, gastrointestinal, and constitutional symptoms. In a retrospective survey conducted in 2000 by Goff and colleagues [3], 77% reported abdominal symptoms (bloating, pain, and increased size); 70% gastrointestinal symptoms (indigestion, constipation, and nausea); 58% symptoms involving pain (abdominal pain, pain with intercourse, and back pain); 50% constitutional symptoms (fatigue, anorexia, and weight loss); 34% urinary symptoms (frequency or incontinence); 26% pelvic symptoms (bleeding, a palpable mass). Goff and colleagues developed a symptom index [8] in which four symptoms were considered significant. These symptoms are bloating/increase in abdominal size, pelvic/abdominal pain, difficulty eating/feeling full quickly, and frequent or urgent urination. In a study by Hamilton and colleagues, symptoms of abdominal distension, urinary frequency, and abdominal pain remained independently associated with a diagnosis of ovarian cancer [22].

Women with ovarian cancer experience symptoms more frequently, severely, and persistently than women without the disease [11]. Olson and colleagues found that bloating, fullness, and abdominal pressure were significantly more likely to be experienced continuously by women with cancer when compared with controls (62% versus 36%) [23]. One of the findings of the current survey is that only 26.4% respondent GPs thought that the patient with ovarian cancer are more likely to have frequent, sudden, and persistent symptoms, with only about 20% saying the symptoms are very frequent (12–30 times a month). These findings suggest a gap in knowledge with regard to symptoms of ovarian cancer.

About one-fifth of the respondent GPs were either unsure or not knowing the importance of family history of breast cancer. In the pathfinder study, 23% of the male GPs and 4% of female GPs were not aware of the importance of the family history of breast cancer [24]. In another questionnaire survey of the GPs referring patients to family cancer clinic for breast/ovarian cancer, it was demonstrated that many GPs need help to recognise which patients to be referred to the family cancer clinic, with one in four referrals of patients with low risk family history [25]. A further study showed that only 26% of the respondent GPs were able to name the three most important criteria used for risk assessment [26].

It is recommended that any woman with symptoms suggestive of ovarian cancer should have a careful pelvic examination [27]. The National Institute for Health and Clinical Excellence (NICE, UK) Referral guidelines for suspected cancer in 2005 suggested that any woman with a palpable abdominal or pelvic mass on examination that is not obviously uterine fibroids or not of gastrointestinal or urological origin should have an ultrasound scan. If the scan is suggestive of cancer, or if ultrasound is not available, an urgent referral should be made [28]. In our survey, male GPs were more likely (*x* ^2^ = 8.5, *P* = 0.01) to feel uncomfortable suggesting vaginal examination. Similarly, in the pathfinder study [24], only 68% GPs performed vaginal examination before referring a patient with suspected ovarian cancer. Many clinicians believe that vaginal examination is a dying skill, uncomfortable, helps only little in terms of diagnosis, and less accurate than ultrasound. There is also increasing fear of patient dissatisfaction compounded by the nonavailability of the chaperone at the time of examination. There is an increasing emphasis on the use of chaperone during intimate examinations by the royal colleges [29], the General Medical Council [30], and the defence organisations. Although the detection rate of ovarian cancer by clinical examination is not very high, a fear to carry out vaginal examinations may result in increase in the number of referrals for pelvic ultrasound, with no obvious benefit. In fact, a large US randomized trial found some harm to women who were screened annually with a transvaginal ultrasound exam and a CA-125 blood test compared with a usual care control group [31]. It is important to note that NICE, UK has since updated the guidance for recognition and initial management of ovarian cancer in 2011, which now suggests that those patients identified by examination to have ascites or abdominal or pelvic mass should be referred urgently [32]. As our survey was carried out before publication of the new NICE guidance, it would be of great interest to repeat the survey to assess the change in practice in primary care in light of the new NICE guidance on ovarian cancer. We aim to carry out a repeat survey as soon as the funding becomes available.

Our study highlights the importance of regular updates and information as a tool to keep abreast with current research evidence. There is a need for regular physician updates with discussion on recent evidence and research to dissipate the knowledge among the primary care clinicians. Gynaecological oncology centres with interested charities should work with primary care clinicians to increase the awareness of risk factors for ovarian cancer and current research in symptom based detection of ovarian cancer amongst health care professionals.

## 5. Limitations

One of the limitations of this survey is the relatively low response rate (27.4%) compared to other postal questionnaire surveys of health care professionals in primary care [33, 34]. Nevertheless, the number of responses we received constituted a large sample size to inform conclusions. Kelley and colleagues [35] also noted in their article that postal questionnaires usually have low response rate of about 20% depending on the content and length of questionnaire. For this reason, a large sample size is required to ensure that the demographic profile of survey respondents reflects that of the survey population and to provide sufficiently large dataset for analysis.

## 6. Conclusion

In spite of the above-mentioned shortfalls, the current questionnaire-based survey has given some very important messages with regard to clinician's perception on knowledge and awareness of ovarian cancer symptoms and risk factors. Majority of the respondent primary care clinicians were well aware of the common symptoms that may suggest an underlying ovarian cancer. However, the relevance of gastrointestinal symptoms such as altered bowel habits, feeling full quickly, and indigestion were not identified. As about 10% of ovarian cancers are genetically linked, it is absolutely vital that all health care professionals are aware of the importance of family history of breast and ovarian cancer. With increasing evidence that almost all patients with ovarian cancer have symptoms, and they report them to their clinicians, the onus is on clinicians to identify and refer the patients urgently in a timely manner. Regular updates would keep the clinicians aware of the ongoing research in the field as new evidence become available more rapidly.

## Figures and Tables

**Table 1:** Responses to detection of ovarian cancer questions (110 responses).

Questions	Yes (%)	No (%)
Ovarian cancer is detected by a smear test	0	110 (100%)
Almost all woman diagnosed with early stage disease are reporting symptoms	103 (93.6%)	7 (6.4%)
Early clinical diagnosis is possible	65 (59.1%)	45 (40.9%)
Women with ovarian cancer are more likely than those with benign conditions to experience very frequent, sudden onset, and persistent symptoms	29 (26.4%)	81 (73.6%)

**Table 2:** Expected frequency of symptoms in ovarian cancer patients (91 responses).

Frequency of symptoms	No (%)
Never	10 (11%)
2-3 times a month	33 (36.3%)
4–12 times a month	30 (33%)
12–30 times a month	18 (19.8%)

Total	91

## Abbreviations

GPs: General Practitioners
NICE: National Institute for Health and Clinical Excellence.
